# HTA capacity building in Asia: towards one goal

**DOI:** 10.1017/S0266462323000405

**Published:** 2023-08-15

**Authors:** Linda Mundy, Guy Maddern

**Affiliations:** 1 School of Public Health, Faculty of Health and Medical Sciences, University of Adelaide, Adelaide, Australia; 2 Chair, HTAi Asia Policy Forum, Discipline of Surgery, University of Adelaide, The Queen Elizabeth Hospital, Woodville South, Australia

**Keywords:** capacity building, technology assessment, health, decision-making, health policy, Asia

## Abstract

**Objectives:**

The aim of the 2022 Health Technology Assessment International (HTAi) Asia Policy Forum (APF) was to discuss experiences and challenges around health technology assessment (HTA) capacity building for both HTA agencies and companies in the Asia region and to identify possible solutions as part of a capacity building roadmap.

**Methods:**

Discussions during the 2022 APF, informed by a pre-meeting survey of HTA agencies and industry attendees from the region, form the basis of this paper.

**Results:**

HTA is an essential element of priority-setting in healthcare; however, the scarcity of skilled technical HTA practitioners is a rate-limiting step in the conduct of HTA. The lack of investment in HTA and the political will to mandate the use of HTA in decision-making may be due to a lack of understanding of the value of the HTA process, and how HTA is interpreted and used in the healthcare decision-making process.

**Conclusions:**

Increased demand for HTA is created when the value of HTA is recognized. HTA capacity-building challenges may be mitigated by educating stakeholders, particularly policymakers, on the value of, and the need to invest in, HTA as a transparent process to ensure equitable access to health care for all. Investigating a means of funding and implementing an HTA intern program between agencies, in partnership with industry, to facilitate a supportive environment to foster HTA skills and knowledge, build capacity or strengthen existing capacity should be a priority.

## Introduction

Health Technology Assessment (HTA) is defined as a multidisciplinary process that uses explicit methods to determine the value of a health technology at different points in its lifecycle. The purpose is to inform decision-making in order to promote an equitable, efficient, and high-quality health system (1). By informing and supporting priority-setting, HTA ensures the efficient use of limited healthcare resources. Strengthening priority-setting by building HTA capacity in the region is critical for the formulation of benefit packages that can deliver equitable, high-quality, and affordable health care for all (2). Although the foundations of HTA infrastructure are in place in many countries in the Asia region, there remains a gap between supply and demand of HTA capacity as the need for HTA grows (3).

Previous APF discussions revealed a lack of capacity in Asia as a barrier to conducting HTA, with HTA agencies in the region struggling to recruit, train and retain skilled HTA practitioners. The lack of HTA capacity was highlighted as a pressing and challenging issue during the COVID-19 pandemic when many COVID-19-related technologies (drugs, vaccines, and interventions) had to be evaluated and approved based on limited evidence within a short timeframe. In addition, a lack of capacity to enable information and data sharing has prevented the use of real-world data to inform decision-making, and in some cases, a lack of technical capacity has limited the ability to assess uncertainty around economic analyses (4;5). Some HTA agencies reported that efforts to build capacity have been negated by the lack of political will to mandate the implementation of HTA in the decision-making process (6).

There appears; however, to be a disconnect between the past experiences of HTA agencies and that of industry APF delegates, who reported in previous APFs that their companies were involved in a range of capacity-building activities in conjunction with HTA agencies in order to support the development of robust evidence generation infrastructure in the region. Many companies reported being involved in the training and development of HTA skills and methodologies using a collaborative approach including investing in infrastructure, advocating the use of databases, conducting early assessments and pilot projects, and supporting universities and think tanks (4).

### What is capacity building?

Capacity building is a complex undertaking (7), where the transfer of knowledge and skills at the local level empowers individuals, and in so doing, improves the effectiveness and sustainability of organizations such as health systems (8). Capacity building should exploit and develop existing local capacities with a locally driven context and agenda, and should be sustained and maintained over time, offering opportunities for ongoing learning and change (9). Strong partnerships with external players, including funding agencies (e.g. the World Bank) or, as in the case of HTA, agencies such as NICE International, can offer support, advice or help create the right external incentives for capacity-building processes. However, local players in whom capacity development is being targeted should be responsible for identifying their needs, then managing and driving the process of change. What capacity building looks like and how it is implemented must be ‘owned’ by local stakeholders, taking into account cultural complexities, in equal partnership with any external players supporting the process (7).

**Figure 1.**
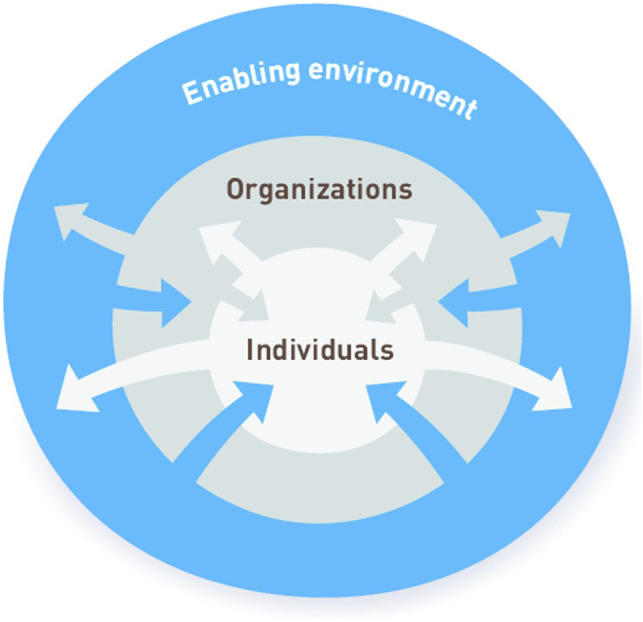
The inter-related three-dimensions of capacity building (10).

Capacity building is multi-tiered, with the individual, organizational, and environmental levels being interconnected and reliant on each other (see Figure 1) (7;11). At the individual level, stakeholders include the “doers”––those individuals who are involved in conducting HTA, including academic and industry researchers, as well as clinicians, patients and carers who provide their lived experiences to ensure the HTA is relevant to the local context. Although the organizational and environmental levels are critical for HTA to be embedded into health systems, capacity building at the individual level is essential for successful HTA, giving individuals the means to increase and improve their technical skills, experience and knowledge in order to conduct, interpret and use HTA effectively (12;13). Networking and collaboration at the individual level is also key for the transfer of HTA knowledge, with individuals who have acquired HTA skills able to mentor others, in so doing, grow capacity (12). HTA capacity building at the organizational level must consider structures, policies, and procedures of HTA, encompassing both within- (e.g. a HTA agency embedded within a university or hospital) and between organization relationships (e.g. HTA agency or university, and the Department of Health (DOH)) (12). Institutional arrangements are important to ensure a credible and transparent assessment process can be established to translate evidence into policy in a local context (3). Developing strong links between HTA organizations and health policy-makers is important, ensuring decision-makers have confidence in the quality of the HTA process and the relevance of the HTA product to the end user (12;13).

Improving policy frameworks at the environmental or system level enables organizations, institutions, and agencies to enhance capacity by addressing economic, political, environmental, legal, and social factors in a coherent and mutually reinforcing fashion. It is especially important that stakeholders at all levels not only understand the value of, and the need for, priority-setting in healthcare but also have the capacity to understand the HTA process and how HTA outcomes are interpreted and used in the healthcare decision-making process (3;12;13).

One of the main priorities of HTA capacity building at the environmental level in a healthcare context means putting in place the political will, governance, and policy structures to support HTA capacity to inform decision-making and in so doing promote an equitable, efficient and high-quality health system (14). A recent survey by Sharma et al. (3) reported that, although most countries in the region had HTA agencies despite a lack of an explicit remit or legislation mandating the use of HTA, the lack of political will and support prevented the institutionalization and widespread integration of HTA into health systems. Political support, as opposed to political interference, was viewed as crucial to drive the translation and adoption of HTA recommendations into policy (3).

Networks, either within a country or between countries, are an important element of capacity building. Networking may be as simple as collaboration between institutions on specific projects or general networking opportunities such as that offered by collaborative organizations like HTAsiaLink, which facilitates countries across the Asia region to share their HTA experiences, learnings and resources, providing opportunities to share technical and methodological know-how (14-17). Importantly, highly valued networks such as HTAsiaLink build interpersonal relationships among member countries, fostering a willingness to collaborate, mutual trust, respect, and open communication (16).

The development of HTA capacity where it is lacking can better inform decision-making enabling the more effective, efficient and equitable use of health resources, which will, in due course, result in better health outcomes for the population as a whole (18). The objective of the 2022 APF was, therefore, to identify the main issues for both industry and HTA-agencies, and to explore possible solutions around capacity building in the Asia region.

## Methods

The tenth APF was held from November 2 to 4, 2022, in Singapore, attended by 21 representatives from 11 not-for-profit organizations (HTA agencies, payers, and health systems), and 25 representatives from 13 for-profit organizations (pharmaceutical, biotech, and medical device companies). To inform discussions, a background paper (19) was developed comprising a literature review that identified the issues and challenges around HTA capacity specific to the Asia region, as well as pre-meeting surveys where both agency and industry attendees of the APF described their experiences of HTA capacity building (Table 1).

**Table 1.** Industry and HTA-agency pre-meeting surveys on capacity building

HTA-agency survey
Is your agency Embedded within the Department of Health Embedded within or have strong links with a university An independent (private sector) agency?
Who funds your HTA activities? Check all that apply Department of Health Other government departments Regulators Non-government organizations (e.g. WHO) Industry/private sector International agencies (e.g. NICE International) Other (please specify)
Who does your agency conduct HTA for? Check all that apply Department of Health Other government departments Regulators Non-government organizations (e.g. WHO) Industry/private sector International agencies (e.g. NICE International) Other (please specify)
What type of technologies does your agency assess? Check all that apply Drugs Medical devices Diagnostic tests Vaccines Surgical procedures Health screening programs Public health programs Other (please specify)
What are the limitations of the HTA infrastructure in your country? Check all that apply Lack of technical expertise to conduct HTA Lack of HTA training Lack of expertise in implementation of HTA Lack of funding for HTA development Lack of political support for using HTA in policy Other (please specify)
As of the beginning of 2022, how many full-time and part-time staff did your agency employ?
Do you think that this staffing level is Below capacity Just right Above capacity
On average, how long do staff stay in your agency? < 1 year 1–3 years >3 years
What are the limitations in respect to retaining HTA staff? Check all that apply Lack of pay Lack of opportunities for promotion Lack of professional development Staff head hunted by private sector/industry Change of career Lack of research opportunities Lack of recognition Stressful work environment Other (please specify)
Of the limitations above, which factor do you think is the most important?
If below capacity, what key skills are you missing? Check all that apply Administration staff HTA specialist––pharmaceuticals HTA specialist––devices HTA specialist––diagnostics HTA specialist––epidemiologist HTA specialist––evidence-based medicine HTA specialist––qualitative Health economist Biostatistician Healthcare policy Regulatory expert Other (please specify)
Thinking only of your staff involved in conducting HTA, what qualifications do they have? Check all that apply Biosciences Medicine Psychology Statistics Pharmacy Post-graduate in HTA-related field (epidemiology, biostatistics etc) Other (please specify)
Of the staff who have an undergraduate qualification, how many have an HTA-related post-graduate qualification (epidemiology, biostatistics etc.)?
Does your agency seek to recruit…… Check all that apply Qualified HTA personnel––domestically trained Qualified HTA personnel––Internationally trained University graduates and train HTA skills internally Entry level staff (no degree) and train HTA skills internally
What HTA training opportunities are available in your agency? Check all that apply Internal training and mentoring Formal post-graduate HTA courses run by local universities Online HTA courses (MOOCs) run by international universities Links with industry active in the region None Other (please specify)
Do these training opportunities adequately cater to the HTA demand in your country?
What networking opportunities do staff at your agency get to undertake to develop links with the HTA community? Check all that apply HTAsiaLink HTAi ISPOR INAHTA Local forums Other
Finally, what do you see as your agency’s greatest challenge and its greatest success in terms of HTA capacity building?
*Industry survey*
Does your company have an internal HTA capacity?
If no, does your company have active HTA engagement with…. Department of Health-based HTA agencies University-based HTA agencies (Asia region) University-based HTA agencies (non-Asia region) Private provider HTA agencies (Asia region) Private provider HTA agencies (non-Asia region) Other (please specify) None
If yes, where is this capacity based? In the Asia region Outside of the Asia region
If yes, how many full-time and part-time staff does your company have dedicated to HTA?
Thinking only of your staff involved in conducting HTA, what qualifications do they have? Check all that apply Biosciences Medicine Psychology Statistics Pharmacy Post-graduate in HTA-related field (epidemiology, biostatistics etc) Other (please specify)
Of the staff who have an undergraduate qualification, how many have an HTA-related post-graduate qualification (epidemiology, biostatistics etc.)?
What HTA training opportunities are available in your company? Check all that apply Internal training and mentoring Formal post-graduate HTA courses Access to online HTA courses (MOOCs) None Other (please specify)
Is your company actively involved in the training and development of HTA skills in the region?
If yes, is this training conducted…. Directly with HTA agencies In conjunction with local Universities Other (please specify)
Does this training consist of developing skills in….(Check all that apply) General HTA (quantitative assessment: safety, efficacy etc) Health economics methodologies Biostatistics methodologies Data collection Data analysis Developing fit-for-purpose registries Qualitative methodologies Patient assistance programs Providing ‘education’ for policy-makers Other (please specify)
In your experience, what are the greatest gaps in HTA in the region? Click all that apply Basic HTA methodology (quantitative assessment: safety, efficacy etc) Health economics methodologies Biostatistics methodologies Policy-maker education (policy development from evidence) Clinician education Public/patient education Regulator education Access to data Other (please specify)
What are the limitations of the HTA infrastructure in the Asia region? Check all that apply Lack of technical expertise to conduct HTA Lack of HTA training Lack of expertise in implementation of HTA Lack of funding for HTA development Lack of political support for using HTA in policy Other (please specify)
What networking opportunities do staff in your company get to undertake to develop links with the HTA community? Check all that apply HTAsiaLink HTAi ISPOR INAHTA Local forums Other (please specify)
Finally, what do you see as your company’s greatest challenge and its greatest success in terms of HTA capacity building in the region?

The APF is designed to promote open and constructive dialogue, without fear or favor. As such, meetings are conducted under the Chatham House Rule in which participants are free to share information obtained during the meeting but the identity or affiliation of the person providing the information cannot be revealed (20). This paper provides the authors’ summary of some of the key messages and main discussion points of APF 2022 and does not necessarily represent the consensus view of those attending the meeting, or those of the organizations they represent.

## Results

The results from the two pre-meeting surveys are summarized below, interspersed with commentary (in italics) from discussions that took place during the 3-days of the APF.

### Summary of the results from the agency survey

**Figure 2.**
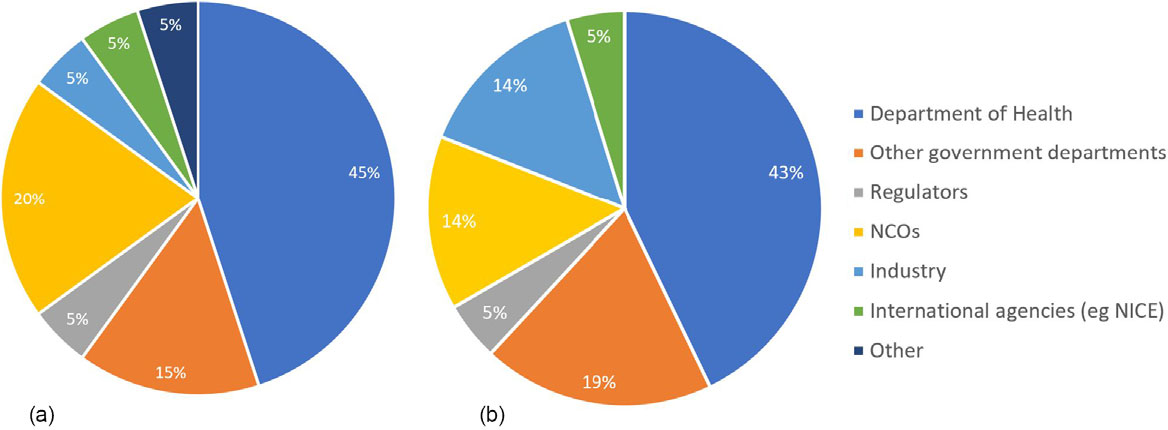
(A) The source of funding for HTA agencies and (B) HTA conducted on behalf of.

Nine of the 11 public sector HTA agency participants responded to the survey in full, representing Cambodia, China, Indonesia, South Korea, Malaysia, the Philippines, Singapore, Taiwan, and Vietnam. Only one agency reported being embedded within a university (Fudan University, Shanghai, China), with the remaining embedded within their respective DOH. Of the eight DOH-embedded agencies, only three reported having strong links with universities: Taiwan, Malaysia, and Indonesia. Four agencies were wholly funded by the DOH, with three conducting HTA only for the DOH: Taiwan, the Philippines, and Singapore. Malaysia, although only funded by the DOH also conducted HTA on behalf of other government departments and regulators. All other agencies were funded in part by the DOH, and all conducted HTA on behalf of the DOH; however, additional funding was received from other sources, and HTA was conducted for these funders (Figure 2).

*Delegates agreed that being embedded within the DOH made it easier to develop and maintain good working relationships, in turn making it easier to provide the right advice to policymakers and have a positive impact on health care delivery.*

When respondents were asked about the limitations of HTA infrastructure in their country, responses were consistent across the region. A lack of expertise in the implementation of HTA was cited by six countries, with five reporting that a lack of technical expertise to conduct HTA and a lack of funding for HTA development are major issues. Interestingly, only Cambodia and Indonesia reported that a lack of HTA training was a limiting factor. A lack of political support for using HTA in health policy decision-making was reported by three countries: Indonesia, Vietnam and South Korea. The Philippines identified a lack of research networks with universities to expand capacity for assessments as a limiting factor, and Vietnam cited a lack of local data to support HTA studies. Singapore noted the main limitation in conducting more HTA evaluations was insufficient human resources rather than a lack of technical expertise. Similarly, Malaysia reported an inadequate number of experts, and that expertise in this highly skilled area needs to be strengthened.

*Delegates agreed that political will is needed to drive increased funding of HTA, with a dedicated training budget that is separate from a ‘core business’ budget.*

**Figure 3.**
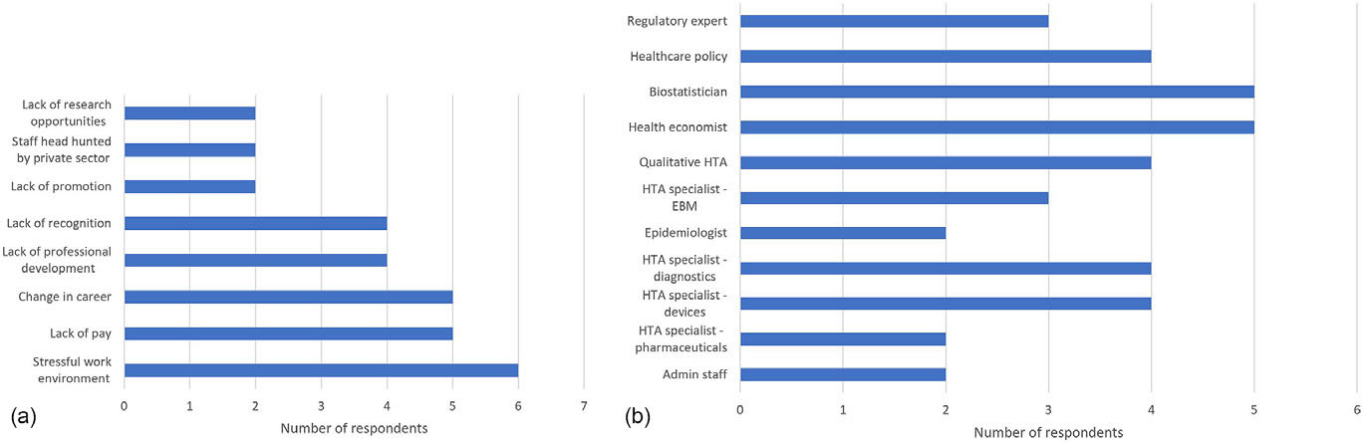
Reported (A) limitations to the retention of HTA agency staff (B) key competencies missing from the respective HTA workforce.

Although staffing levels varied across the region, most countries reported staffing levels below capacity (77.8 percent). Only Taiwan and Vietnam reported appropriate staffing levels. Retainment of staff didn’t appear to be an issue, with most countries reporting that staff were employed for more than 3 years, with only Taiwan and the Philippines reporting an average employment period between 1–3 years. When asked to think about the factors that made retention of HTA staff difficult, the most common reason cited by six agencies (66.7 percent) was a stressful work environment, with insufficient staffing levels contributing to increased workloads, leading to a lack of opportunities for research and staff appearing to be stressed at work. A lack of remuneration and a change in career were the next most common reasons for staff turnover (5/9, 55.6 percent). Interestingly, only Taiwan and Singapore reported a loss of HTA staff to the private sector (Figure 3A).

*Delegates raised concerns around the growing complexity of conducting HTA with the increasing number of evaluations for technologies such as gene and cell-based therapies, and genetic testing. Not only do these evaluations require adaption to existing HTA methodologies but they also require more time, leaving HTA agencies feeling pressured to complete complex assessments in short time frames.*

When asked to identify the key competencies and skills missing from their agency, responses varied widely (Figure 3B). Singapore reiterated that, overall, they had the right skill set balance but just not enough human resources to cope with increasing workloads. Cambodia reported deficiencies in all areas of HTA, closely followed by Vietnam. The most common “missing” skill sets reported were health economists and biostatisticians (5/9, 55.6 percent). Shanghai reported that in addition to administrative and staff qualified in healthcare policy, that medical informatics was a skill that they would like to recruit.

Many staff involved in HTA have undergraduate degrees in disciplines such as the biosciences, medicine or pharmacy, and then use this skill set in addition to gaining post-graduate qualifications in an HTA-related field. All agencies except for Cambodia reported that their staff had post-graduate qualifications in an HTA-related field such as epidemiology or biostatistics. Malaysia also reported staff members qualified in hospital administration and as public health physicians. Countries with large agencies such as South Korea (125 researchers), Singapore (80 technical and non-technical staff) and Taiwan (40 staff) reported high rates of HTA post-graduate qualifications in their full-time staff (ranging from 25–80 percent). Demonstrating extremes of capacity, Shanghai reported that all faculty staff had a PhD qualification, whilst Cambodia had no staff, and the Philippines and Vietnam had low numbers (2 each) of staff with an HTA-related post-graduate qualification. Smaller agencies such as Indonesia also reported a high proportion (5/10, 50 percent) of their staff had appropriate post-graduate HTA qualifications. When thinking about filling these gaps in competencies, overwhelmingly, agencies, with the exception of Vietnam, sought to recruit university graduates, looking to train them in HTA skills internally. In addition to entry-level staff, with no degree to train in HTA internally, most agencies (66.7 percent) also seek to recruit domestically and internationally trained HTA personnel.

With upskilling at the individual level identified as an important component of capacity building, respondents were asked about training opportunities offered to staff. Only Cambodia could not offer any HTA training, with all other agencies offering internal training and mentoring. Most agencies offered access to post-graduate courses run by local universities, such as a Master of Public Health, evidence-based medicine, and health economics. In addition, many agencies have embraced the use of massively open online courses (MOOCs), many of which are offered for free. Only South Korea and Indonesia made use of training opportunities offered by industry active in the region. The Philippines, Cambodia, Shanghai, and Indonesia reported that current access to training opportunities did not adequately address the demand for HTA in their country. It should be noted that agencies such NECA Korea, HITAP Thailand and Singapore’s ACE not only offer internal HTA training but also play a key role in providing publicly accessible offline and online HTA courses in addition to sharing HTA method manuals.

*Various HTA training methods were discussed, with MOOCs being identified as a good way to build capacity, deliver skills education and share knowledge. Although MOOCs are relatively cheap and easy to deliver, concerns around whether they were fit-for-purpose were raised as they don’t tend to consider the local context, especially funding arrangements. The pros and cons of requiring staff to hold post-graduate HTA qualifications was also discussed. Consensus was that in the fast-paced health technology world, an agile workforce armed with open thinking, engagement, a natural curiosity, and a willingness to work hard and learn new skills was viewed as more important than one with purely academic know-how. However, on-the-job-training was viewed as no longer sustainable for a fit-for-purpose HTA workforce. Governments need to invest in training by funding education through universities, with consideration given to targeted scholarships. Opportunities for industry to partner with universities should be explored.*

As previously discussed, networking and collaboration have been identified as key to facilitating the transfer of knowledge and skills, enabling mentoring opportunities, and in so doing, growing capacity. Agencies in the region clearly value networking, with HTAsiaLink and HTAi foremost among networking opportunities reported by eight of the nine agencies (88.9 percent), with the Professional Society for Health Economics and Outcomes Research (ISPOR) also highly regarded for networking (66.7 percent).

*Although not core-business, discussions during the APF identified giving staff opportunities to write peer-reviewed publications was important to get feedback from colleagues on methodology and build professional development. Publications can also impact on policy, provide another way for countries to ‘collaborate’ by sharing their experiences and may provide opportunities for agencies to collaborate with industry to present both sides of the HTA coin.*

### Summary of the results from the industry survey

Ten of the 13 participants from industry responded to the survey; however, only nine answered in full. Of these, four are device manufacturers, four are pharmaceutical companies, and one markets both pharmaceuticals and vaccines. All companies reported having an internal HTA capacity, mostly based in the Asia region (60 percent), with only one company reporting a more global-focused HTA capacity, whilst three companies reported both a global and Asia regional-based capacity.

Due to global team structures, many companies found it difficult to quantify the number of staff dedicated to HTA with staff having multiple functions within the company, supporting activities such as market access as well as HTA activities. Of the six companies that could quantify the number of staff dedicated to HTA activities, numbers ranged from 2–3 full-time equivalent in smaller companies, up to more than 30 employees in larger global companies, many of whom would have a focus on particular countries in the region. Regardless of their undergraduate qualification (biosciences, medicine, pharmacy or statistics), all companies reported that all staff involved with HTA activities had an HTA-related post-graduate qualification, such as public health, epidemiology, health economics or biostatistics.

HTA training opportunities of some kind were offered by all companies, especially improving HTA capacity by delivering internal training and mentoring as a minimum. Most companies (70 percent) supported their staff financially and with time off to access online HTA courses, as well as formal post-graduate HTA courses and short courses such as those run by ISPOR, demonstrating a commitment to build and invest in HTA. This commitment is also reflected by most companies (70 percent) being involved in the training and development of HTA skills in the region. How this training is delivered may offer future opportunities for collaboration, as no companies reported having direct links with HTA agencies but rather conducted training in conjunction with local universities. This training covered a wide gamut of HTA skills, including general quantitative HTA, health economics (not just cost-effectiveness but also the articulation of value), biostatistics, as well as data (especially real-world data) collection and analysis. Interestingly, four companies were involved in providing HTA education for policy-makers, whilst others were interested in developing skills around fit-for-purpose registries (2/9) as well qualitative methodologies and patient assistance programs.

Industry also recognized the important role that networking can play in developing collaborations and developing skills. Overwhelmingly, industry respondents provide their staff access to HTAi (7/9), ISPOR (8/9) and local forums (7/9) to develop links with the HTA community.

*Delegates agreed that industry and agencies had many similarities, with both wanting to provide patient access to new health technologies. Collaboration between industry and agencies during the submission process may result in better market access outcomes for industry and improved efficiencies for agencies. Early and continuous two-way dialogue and exchange of ideas between industry and agencies throughout the assessment process and implementation should be encouraged, with resource sharing of training materials via a neutral platform, with universities potentially playing an important role in this space.*

**Figure 4.**
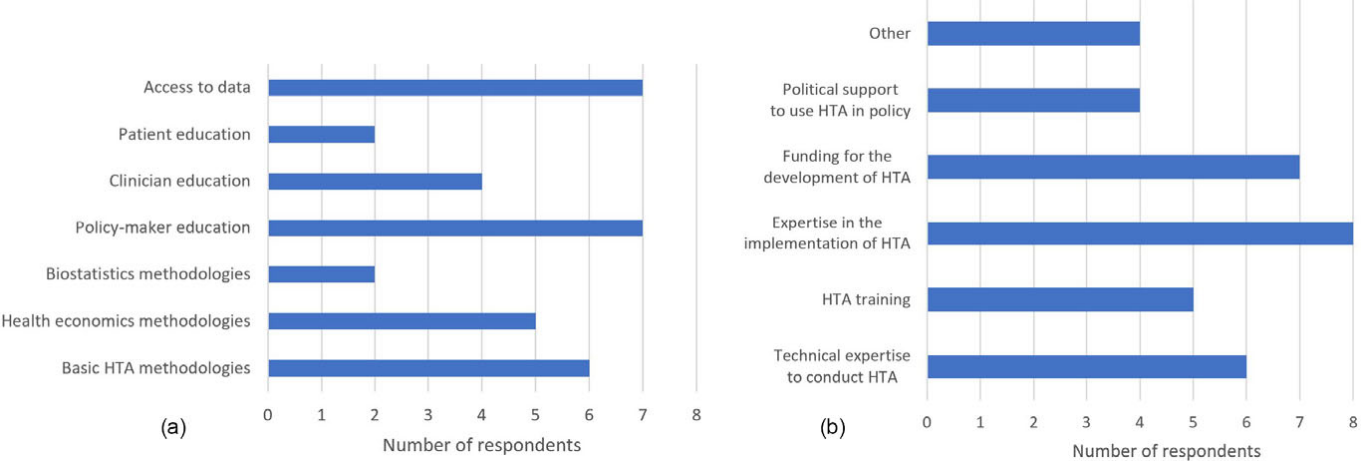
Factors identified by industry respondents (A) HTA skills needed in the region (B) elements of HTA infrastructure missing in region.

When asked to think about where the greatest gaps in HTA lie in the region, overwhelmingly respondents nominated access to data and policy-maker education (7/9, 77.8 percent) as the biggest issues, closely followed by basic HTA and health economic methodologies (Figure 4A). The applicability of evidence for decision-making was also raised, which ties in with the concern around understanding that alternatives to cost per quality-adjusted life year (QALY) may be more suitable to use in countries where there is limited access to data.

*Delegates agreed that educating political stakeholders on the implications of HTA recommendations would enable them to engage meaningfully with the process. There is a real need to build local capacity with local training programs, with training tailored to the targeted audience––patients, clinicians and policy-makers. Broader engagement will encourage ecosystem thinking rather than the creation of silos.*

Respondents were then asked to identify the most important elements they perceived to be currently lacking in HTA infrastructure in the region (Figure 4B). Interestingly, most respondents identified a lack of expertise in the implementation of HTA over the lack of funding and technical expertise to conduct HTA. Other issues raised included the misuse of HTA for cost-cutting purposes, reinforcing the lack of policy-maker education. Human resource capacity was also identified as a problem, with the lack of HTA personnel resulting in a lack of capacity to produce product reviews in a timely manner.

## Discussion

From discussions during the APF, it became clear that both industry and agencies have similar issues with the recruitment, training, and retention of staff who understand and can conduct HTA. Many in industry felt that considerations of HTA in the development of a healthcare technology was secondary to, and not as important as sales and marketing roles. Many individuals in industry and agencies felt that there was a lack of career opportunities in HTA, with staff looking for opportunities to grow and gain recognition, especially in the face of HTA becoming more complex and demanding (increasing) workloads. Both sectors employ staff with diverse educational backgrounds, then provide on-the-job training as it is rare to employ staff with HTA experience. Staff need multiple skill sets and continuously need to learn and develop new skills.

Internal and external training should consider the different perspectives of HTA––HTA developed by academia, adopted by industry, and used by reimbursement bodies for decision-making. There are cross-pollination opportunities for industry, government, and academia to collaborate. By working in partnerships, these stakeholders need to establish a structure or framework to build capacity that can benefit all players, by addressing needs and gaps in order to achieve patient access to new healthcare technologies. To this end, strong leadership is needed, with governments recognizing HTA capacity as a whole-of-system issue by providing adequate funding.

After discussing ways in which the APF could promote capacity building in the region, delegates agreed that HTAi should champion existing networks to develop the potential of young researchers by funding a scholarship or intern program, giving financial support for a candidate from one country to visit another country’s HTA agency for a 2-month period. Fellows should already be enrolled in a course of study aligned with HTA and present their experience at HTAi’s annual meeting. In addition, beneficiaries would be expected to return to their home countries to share their acquired knowledge and skills, which would represent a true return on investment. Malaysia, Taiwan and Thailand have all reported good outcomes from similar internships; however, it was noted that programs such as this need a degree of planning due to issues of confidentiality.

## Conclusions

Increased demand for HTA is created when the value of HTA is recognized. HTA capacity-building challenges may be mitigated by educating all stakeholders, particularly policymakers, on the value of, and the need to invest in, HTA as a transparent process to ensure equitable access to health care for all. Investigating a means of funding and implementing an HTA intern program between agencies, with the participation and input from industry, in order to facilitate a supportive environment to foster HTA skills and knowledge, build capacity, or strengthen existing capacity should be a priority.
